# The Human Herpesvirus-7 (HHV-7) U21 Immunoevasin Subverts NK-Mediated Cytoxicity through Modulation of MICA and MICB

**DOI:** 10.1371/journal.ppat.1002362

**Published:** 2011-11-10

**Authors:** Christine L. Schneider, Amy W. Hudson

**Affiliations:** Department of Microbiology and Molecular Genetics, Medical College of Wisconsin, Milwaukee, Wisconsin, United States of America; Oregon Health Sciences University, United States of America

## Abstract

Herpesviruses have evolved numerous immune evasion strategies to facilitate establishment of lifelong persistent infections. Many herpesviruses encode gene products devoted to preventing viral antigen presentation as a means of escaping detection by cytotoxic T lymphocytes. The human herpesvirus-7 (HHV-7) U21 gene product, for example, is an immunoevasin that binds to class I major histocompatibility complex molecules and redirects them to the lysosomal compartment. Virus infection can also induce the upregulation of surface ligands that activate NK cells. Accordingly, the herpesviruses have evolved a diverse array of mechanisms to prevent NK cell engagement of NK-activating ligands on virus-infected cells. Here we demonstrate that the HHV-7 U21 gene product interferes with NK recognition. U21 can bind to the NK activating ligand ULBP1 and reroute it to the lysosomal compartment. In addition, U21 downregulates the surface expression of the NK activating ligands MICA and MICB, resulting in a reduction in NK-mediated cytotoxicity. These results suggest that this single viral protein may interfere both with CTL-mediated recognition through the downregulation of class I MHC molecules as well as NK-mediated recognition through downregulation of NK activating ligands.

## Author Summary

The long coevolution of herpesviruses with their hosts has resulted in the development of a diverse array of viral immune evasion strategies and host counter-strategies. The identification of viral proteins that impair the function of cellular immune-recognition receptors has proven fertile ground for the discovery of fundamental concepts in immunology and cell biology. While the cytomegaloviruses have demonstrated an extraordinary array of immunoevasive tactics, little is known about the immunoevasive strategies of the closely-related human herpesvirus-7 (HHV-7). We have previously demonstrated that the U21 gene product from HHV-7 likely interferes with viral antigen presentation to cytotoxic T cells by rerouting class I major histocompatibility molecules to lysosomes for degradation. In addition to the host's cytotoxic T cell response, virus infection also induces the expression of Natural-Killer (NK) activating ligands, alerting cytotoxic NK cells to identify and kill virus-infected cells. Here we describe a novel function for the same viral protein - U21 - in interfering with NK cell recognition. Our findings provide the first indication that HHV-7, too, may have found it necessary to strategize mechanisms of NK escape.

## Introduction

Human herpesvirus-7 (HHV-7) is a T-lymphotrophic beta-herpesvirus, most closely related to human herpesvirus-6 (HHV-6) and human cytomegalovirus (HCMV). HHV-6 and -7 share many biological properties: HHV-6 and -7 possess genomes that are almost entirely colinear, and both HHV-6 and -7 can cause the formation of giant multinucleated cells in culture, features reminiscent of those seen in HCMV infection *in vitro*. Primary infection with either of these viruses results in a short febrile illness, and more than 90% of adults are seropositive for both HHV-6 and HHV-7 [1].

Like all herpesviruses, HHV-7 remains latent or establishes persistent lifelong infections in its host. In so doing, herpesviruses have evolved numerous strategies to evade immune detection. Most herpesviruses, including HHV-7, have evolved mechanisms to interfere with viral antigen presentation by class I MHC molecules (for review see [2]–[4]). Although preventing surface expression of class I MHC molecules may be an effective means of escaping CTL detection, the absence of class I products from the cell surface may render the host cell susceptible to Natural Killer (NK) cell attack (for review, see [5]).

Activation of NK cells is regulated by the balance of inhibitory and activating signals received through cell surface NK receptors (for review, see [6], [7]). NK inhibitory receptors bind to classical and non-classical class I MHC molecules. NK activating receptors bind to NK activating ligands, some of which are structurally similar to class I MHC molecules. When an NK cell encounters a potential target cell, it is thought to integrate the activating and inhibitory signals it receives; if activating signals prevail, the NK cell can then directly kill its target. In response to microbial infection or other cell stressors, cells can increase the surface expression of NK activating ligands, improving the likelihood that NK cells recognize and kill cells that become harmful. Viral strategies to remove inhibitory ligands (class I MHC molecules) from the cell surface of an infected cell might further skew the balance in favor of NK killing. Not surprisingly, viruses have also evolved counter-strategies to interfere with NK engagement (for review, see [8], [9]). For example, presumably to escape NK detection, several viruses selectively downregulate HLA-A and HLA-B locus products, while leaving HLA-C, -E, and other non-classical class I MHC molecules at the plasma membrane as inhibitory ligands for NK cell receptors (for review, see [5], [10]).

**Figure 1:**
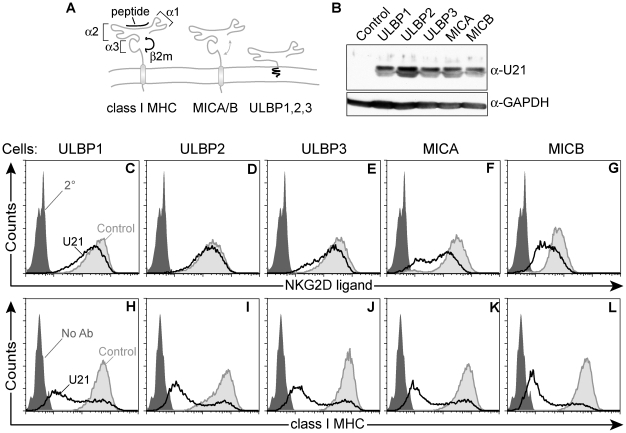
Generation of stable cell lines expressing NK ligands and U21. (A) Schematic representation of class I MHC molecules and NKG2D ligands, depicting α1, α2, and α3 domains of class I MHC molecules, and their overall structural similarity to MICA/B and ULBP molecules. (B) Cell lysates (20 µg) immunoblotted with an antibody directed against U21 or GAPDH, as indicated. Flow cytometric analysis of U373 cells expressing NK ligands or NK ligands and U21. Cells were labeled with antibodies directed against (C) ULBP1 (m295), (D) ULBP2, (E) ULBP3, (F) MICA, and (G) MICB (m360) followed by a PE-conjugated secondary antibody, or with FITC-W6/32 (class I MHC) (H-L). Unlabeled cells (H-L) or cells labeled with the secondary antibody alone (C-G) are indicated by the filled dark gray trace. Control cells labeled with antibodies directed against each NK ligand (C-G) or class I MHC molecules (H-L) are indicated by the filled light gray traces. U21-expressing cells labeled with antibodies directed against each NK ligand (C-G) or class I MHC molecules (H-L) are indicated by the black traces.

In addition to the selective downregulation of NK-inhibitory HLA molecules, another strategy employed by viruses to escape NK engagement is the downregulation of NK activating ligands from the cell surface. For example, the HCMV immunoevasin UL16 was found to bind to two members of a family of cellular proteins termed UL16-binding proteins, or ULBPs [11]. UL16 was also found to associate with a protein called MICB, for MHC class I chain-related protein family [11]. Both the MICs and the ULBPs are activating ligands for the same NK activating receptor, NKG2D, and both MICs and ULBPs share structural similarity with class I MHC molecules (see schematic depicting the general structure of these ligands, Figure 1, panel A) [12]–[14]. HMCV UL16 binds to ULBP1, ULBP2, ULBP6 (RAET1L) and MICB and retains these activating ligands intracellularly, reducing NK recognition of HCMV-infected cells [15]–[18]. Indeed, the NKG2D ligands are frequent targets of viral immunoevasins, underscoring the importance of these ligands in anti-viral immunity. Both murine and human cytomegaloviruses encode multiple immunoevasins that affect NK activating ligands. In addition to UL16, HCMV UL142 retains MICA in the Golgi [19], and MCMV m145, m152, and m155 impair the cell surface expression of murine NKG2D ligands [20]–[23]. Adenovirus E3/19K can also sequester MICA and MICB intracellularly [24], and the KSHV K5 ubiquitin ligase promotes the degradation of MICA and MICB [25], [26]. In addition, HCMV, KSHV, and EBV all encode microRNAs that target MICB mRNA, reducing its expression [27].

We have shown previously that HHV-7 encodes a gene product, U21, that can associate with and affect the surface expression of all classical class I MHC gene products (HLA-A, -B, and -C) by rerouting them to lysosomes, where they are degraded [28]. U21 can also bind to and downregulate the non-classical class I MHC gene products (HLA-E and -G)[28]. Such a comprehensive downregulation of NK-inhibitory class I molecules should shift the balance of ligands toward one that would activate NK cells, alerting them to HHV-7 infection. For HHV-7 to succeed, then, it must also encode a means to escape engagement by NK cells.

Because U21 can associate with and affect the surface expression of such a wide variety of class I MHC gene products, we hypothesized that U21 might also affect the structurally-related activating NKG2D ligands. Class I MHC molecules are composed of three domains, α1, α2, and α3, and they assemble with a light chain, β_2_-microglobulin (β_2_m), and peptide. Like class I MHC molecules, MICA and MICB are also type I membrane proteins that contain α1, α2, and α3 domains, but they do not associate with β_2_m or peptide (see schematic, Figure 1, panel A) [29], [30]. The NKG2D ligands share structural homology, but little amino acid identity with class I MHC molecules; MICA, for example, shares an average of only ∼29% amino acid identity with the HLA-A2 class I heavy chain [11]. The ULBP proteins possess α1 and α2 domains, but lack an α3 domain, and rather than transmembrane domains, ULBP1–3 are GPI-anchored proteins [11].

Here we show that U21 can associate with the NKG2D ligand ULBP1 and redirect it to lysosomes for degradation. U21 can also reduce the surface expression of two other NKG2D ligands, MICA and MICB, resulting in protection from NK cytotoxicity.

## Results

### U21 reduces the surface expression of MICA and MICB

Constitutive levels of NKG2D ligands are low in most cells, thus, to examine the effect of U21 upon the surface expression of the NK ligands, we generated U373 astrocytoma cell lines individually stably expressing ULBP1, ULBP2, ULBP3, MICA, or MICB, using retrovirus-mediated gene transfer. We then stably expressed U21 in each of the NK-ligand-expressing cell lines. Each cell line expressed similar levels of U21, as assessed by immunoblot (Figure 1, panel B). We next investigated the effect of U21 expression on the surface expression of each NKG2D ligand using flow cytometry. U21 expression resulted in a slight downregulation of ULBP1 and ULBP3 from the cell surface, while surface levels of ULBP2 were essentially unchanged (Figure 1, panels C - E, compare light gray shaded traces to black traces). The NK ligands MICA and MICB, however, were more markedly reduced by U21 (Figure 1, panels F and G). Similar results were seen in U21-expressing K562 cells, as discussed later.

U21 also binds to and reroutes class I MHC molecules to lysosomes, reducing their presence at the cell surface. When we examined U21's effect upon the surface expression of endogenous class I MHC molecules in each of the NK ligand-expressing cell lines, endogenous class I molecules were more effectively downregulated by U21 than were any of the NK ligands (Figure 1, panels H-L, compare light gray shaded traces to black traces). Of note, the surface expression of endogenous class I MHC molecules is similarly affected in each of the cell lines, with the majority of U21-expressing cells showing reduced surface class I levels, and a smaller population of cells exhibiting normal surface levels of class I molecules. This broad distribution reflects the variable levels of U21 expression among individual cells; in general, we find that cells expressing higher levels of U21 possess less class I MHC, ULBP1, or MIC on their cell surface (Figure S1). The similar pattern of surface class I MHC downregulation within each population of cells serves as a second, indirect measure of the similar levels of U21 expression in each of the stable cell lines. These results suggest that, in addition to class I MHC molecules, U21 may also impair the surface expression of the NKG2D ligands MICA and MICB. To a far lesser extent, U21 affects the surface expression of the ULBPs.

### U21 redirects ULBP1 to a lysosomal compartment

**Figure 2:**
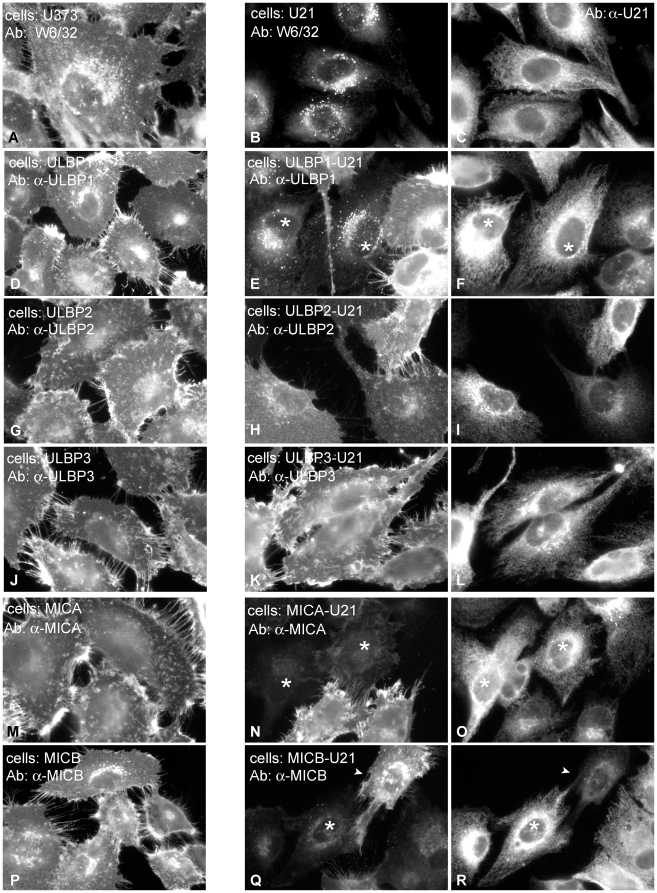
U21 reroutes ULBP1 and MICB to a punctate perinuclear compartment. Left panels: U373 cells (A), and U373 cells expressing each NK ligand and control vector were immunolabeled with antibodies directed against properly-folded class I MHC molecules (W6/32)(A), or against ULBP1 (m295)(D), ULBP2 (G), ULBP3 (J), MICA (M), or MICB (m360) (P), as noted. Middle and right panels: Cells expressing each NK-ligand and U21, as indicated, were double-labeled with antibodies directed against class I MHC molecules (B), ULBP1 (E), ULBP2 (H), ULBP3 (K), MICA (N), or MICB (Q) and anti-U21 (panels C,F,I,L,O, and R). Asterisks in panels E and F denote cells expressing U21 (F) that also exhibit relocalization of ULBP1. Asterisks in panels N, O, Q, and R indicate cells expressing U21 (O and R) that also exhibit reduced labeling of MICA or MICB. The arrowheads in panels Q and R denote a cell with low apparent levels of U21 (R) and normal surface expression of MICB.

U21 expression results in a dramatic steady-state redistribution of class I MHC molecules from the cell surface to the lysosomal compartment (Figure 2, panels A and B). Although U21 accompanies class I MHC molecules to lysosomes, and both molecules are degraded there, U21 does not colocalize with class I MHC molecules in lysosomes [30]. Instead, U21 is localized in the ER/Golgi (Figure 2, panel C, [31], [32]). Explanations for this phenomenon include the possibility that U21 may be more sensitive to lysosomal proteases, such that we are not able to visualize a concentration of U21 in lysosomes. However, incubation in lysosomal protease inhibitors does not significantly alter the steady-state distribution of U21. It is possible the epitopes recognized by our antibodies, all located within the cytoplasmic tail of U21, may become masked upon U21's arrival in lysosomes, accounting for our inability to visualize U21 in lysosomes. Perhaps most likely is the possibility that U21 exists in far greater abundance in the ER and Golgi, such that we cannot see U21 in lysosomes over the brilliant ER/Golgi labeling. We have previously discussed this differential localization of U21 with its client class I MHC molecules in greater detail [30].

**Figure 3:**
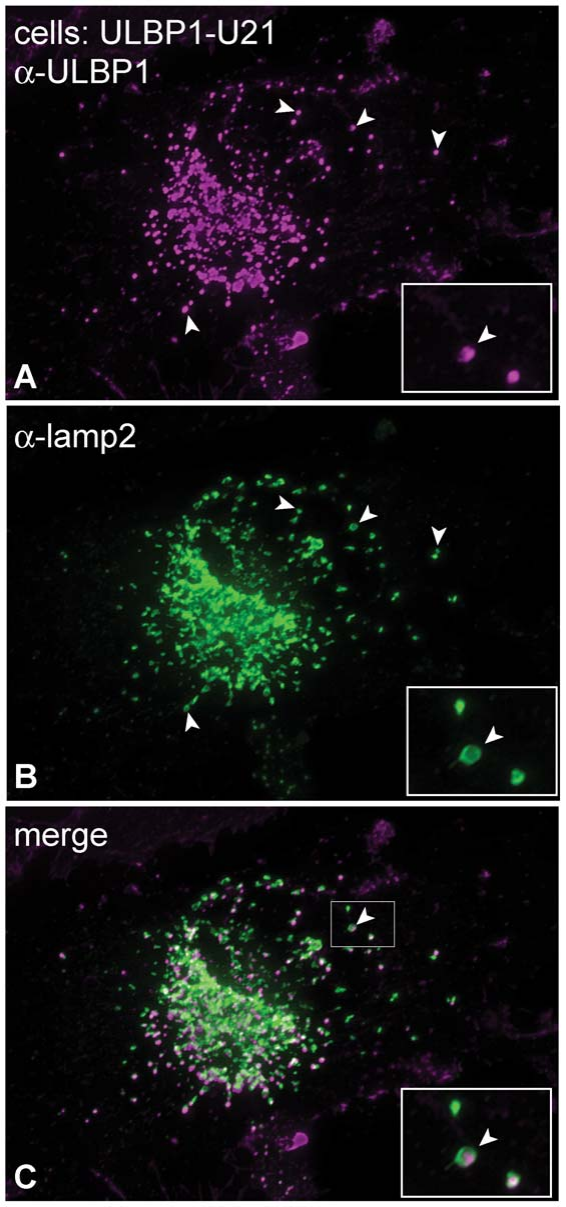
ULBP1 colocalizes with the lysosomal marker lamp2. (A-C) U373 cells expressing ULBP1 and U21 were double-labeled with antibodies directed against ULBP1 (magenta) and lamp2 (green). The merged image is shown in panel C. The arrowheads denote single coincident puncta, and the boxed regions in the bottom right corner of each panel are enlargements of the smaller boxed region with arrowhead in panel C.

Because U21 reduced the surface expression of ULBP1, ULBP3, MICA, and MICB, we next assessed whether expression of U21 might lead to relocalization of the NK ligands within the cell, just as it does for class I MHC molecules. Like endogenous class I MHC molecules, in the absence of U21, the NK-ligands were localized primarily on the plasma membrane, with some labeling of the Golgi, as the molecules traverse the biosynthetic pathway (Figure 2, left panels). U21 expression did not result in appreciable relocalization of ULBP2 and ULBP3, (Figure 2, panels G-L), even though U21 expression resulted in slight reduction of ULBP3 surface expression (Figure 1, panel E). MICA and MICB, on the other hand, seemed to disappear in cells expressing U21 (Figure 2, panels N,O and Q,R asterisks). In cells expressing U21 (Figure 2, panel F), ULBP1 was relocalized to a punctate compartment resembling U21-relocalized class I MHC molecules (Figure 2, compare panels E (asterisks) to B). Redistribution of ULBP1 molecules correlated with the level of U21 expression in the cell; in Figure 2, panels E and F show a population of cells exhibiting heterogeneous expression of U21. In cells with intense U21 labeling (asterisks), ULBP1 punctae are more readily apparent. We therefore examined whether ULBP1 in U21-expressing cells was localized in a lysosomal compartment. Double-label immunofluorescence microscopy showed colocalization between ULBP1 and the lysosomal membrane protein lamp2 in U21-expressing cells (Figure 3).

### U21 binds to ULBP1 and redirects it to lysosomes for degradation

In cells expressing U21, class I MHC molecules are degraded in a lysosomal compartment [32]. Since the relocalization of ULBP1 resembled the relocalization of class I MHC molecules, we next asked whether U21 expression also resulted in the lysosomal degradation of ULBP1, using pulse-chase analysis of ULBP1 in the presence of lysosomal protease inhibitors. Stabilization of a protein in the presence of lysosomal protease inhibitors would suggest a role for lysosomal proteases in the turnover of that protein.

**Figure 4:**
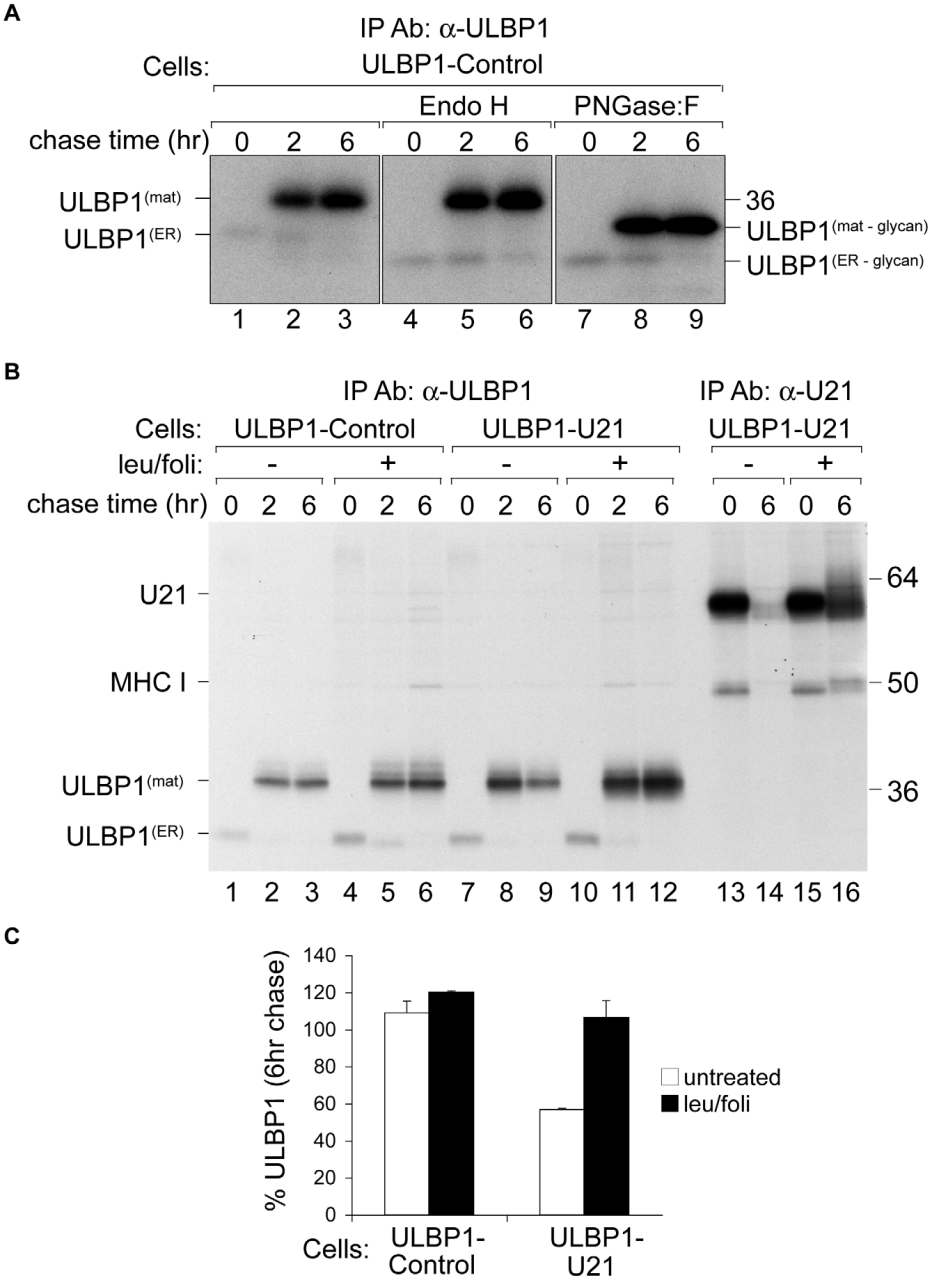
U21 targets ULBP1 for lysosomal degradation. (A) U373 cells expressing ULBP1 were pulse-labeled for 15 minutes and chased for 0, 2, or 6 hours. ULBP1 was recovered from Triton X-100 lysates with anti-ULBP1 (m295) and treated with either Endo H or PNGase:F. Migration of ULBP1 with and without its N-linked glycan are indicated, as is the approximate position of the 36 kDa molecular weight marker. (B) Cells, as indicated, were pulse-labeled for 15 minutes, chased for 0, 2, and 6 hours and either ULBP1 (m295) or U21 were recovered from Triton X-100 lysates. Where indicated, cells were treated with leupeptin (leu) and folimycin (foli). Migration of U21, class I MHC heavy chain (MHC I), and mature (ULBP1^mat^) and immature (ULBP1^ER^) forms of ULBP1 are indicated, as are the approximate positions of molecular weight markers. (C) As a measure of the stability of ULBP1, the percentage of ULBP1 remaining after the 6 hr chase was compared to the percentage of ULBP1 remaining after the 2 hr chase point. The data represent the quantification from three independent experiments (n = 3). Error bars indicate the standard deviation.

We first performed pulse-chase analysis of ULBP1 in the absence of U21. After a 15-minute pulse label of ULBP1-expressing U373 cells, ULBP1 migrated at approximately 31 kDa (Figure 4, panel A, lane 1). At the 2- and 6-hour chase points, ULBP1 migrated more slowly, at ∼37 kDa (Figure 4, panel A, lanes 2 and 3). ULBP1 possesses a single N-linked glycosylation consensus site, thus the ∼6 kDa increase in ULBP1 at the later chase points is either the result of additional modifications to its single predicted N-linked glycan, or possibly O-linked glycosylation.

Surprisingly, we recovered ∼5-fold less labeled ULBP1 immediately following the pulse label (0 hr chase) than after the 2- and 6-hour chase periods (Figure 4, panel A, compare lanes 1 to 2 and 3). One possible explanation for the difference in recovery of ULBP1 is that the ULBP1 monoclonal antibody can more easily recognize ULBP1 after it has moved beyond the ER and Golgi; the immunoprecipitations were performed under non-denaturing conditions, thus it is possible that the epitope recognized by the anti-ULBP1 antibody was partially masked while ULBP1 was in the ER, perhaps as a result of protein complex formation during addition of the GPI anchor. Protein complex formation during addition of the GPI anchor might also explain why we observe no Endo H-sensitive GPI-linked UBLP1 after the pulse label. Alternatively, antibody recognition may become enhanced by modifications to the N-linked glycan that occur in the Golgi compartment or structural changes that occur after addition of the GPI anchor.

To determine whether the size difference between the two species of ULBP1 seen in lanes 1 and 2 was solely attributable to modifications to its predicted N-linked glycan, we digested the immunoprecipitated proteins with either endoglycosidase H (Endo H) or peptide N-glycosidase:F (PNGase:F). Endo H cleaves N-linked glycans found on proteins in the ER and early Golgi. Glycoproteins that have progressed beyond the medial Golgi become resistant to digestion with Endo-H, thus resistance to Endo H can be used to follow the movement of a protein through the secretory pathway. After the 15 minute pulse label, Endo H digestion of ULBP1 resulted in a more rapidly migrating polypeptide of ∼28 kDa, consistent with its predicted molecular weight in the absence of N-linked glycans (Figure 4, panel A, compare lanes 1 and 4). This Endo H-sensitive form represents newly-synthesized ULBP1 present in the ER or early Golgi. At the later chase points, the 37 kDa form of ULBP1 was resistant to Endo H digestion, suggesting that this form of ULBP1 has progressed beyond the medial Golgi (Figure 4, panel A, lanes 5 and 6).

PNGase:F cleaves N-linked glycans from all polypeptide chains, regardless of oligosaccharide processing within the secretory pathway. After the 15 minute pulse label, PNGase:F digestion of ULBP1 resulted in a polypeptide of ∼28 kDa, co-migrating with the 28 kDa Endo H-digested form (Figure 4, panel A, compare lanes 7 and 4). If the slower migration of the 37 kDa form of ULBP1 were solely the result of N-glycosylation, the mobility of the products of Endo H and PNGase:F digestion should be the same – migrating at 28 kDa. Instead, the 37 kDa Endo H-resistant form of ULBP1 was reduced to a polypeptide of ∼31 kDa after PNGase:F digestion, suggesting a separate post-translational modification to ULBP1 (Figure 4, panel A, lanes 8 and 9). ULBP1 is a GPI-anchored protein [11]. It is possible that the slower mobility of ULBP1 may reflect the addition of the GPI anchor. However, since glypiation occurs in the ER, before glycoproteins become Endo H-resistant, we should observe the 31 kDa Endo H-sensitive GPI-linked ULBP1 after the initial pulse label, and we do not. It is also possible that the PNGase:F-resistant form of ULBP1 is O-glycosylated.

Having established the normal trafficking of ULBP1 in the absence of U21, we next performed pulse-chase experiments in U21-expressing cells. To investigate whether U21 expression resulted in the destabilization of ULBP1, we performed pulse-chase experiments in the presence of the lysosomal protease inhibitor leupeptin, and the vacuolar H^+^-ATPase inhibitor folimycin. In cells lacking U21, ULBP1 appeared stable throughout the 6 hr chase period, and lysosomal inhibitors did not significantly increase its stability (Figure 4, panel B, compare lanes 2 and 3 to 5 and 6). In cells expressing U21, however, we recovered fewer labeled ULBP1 molecules after the 6-hour chase period, and in the presence of lysosomal inhibitors, ULBP1 was stabilized (Figure 4, panel B, compare lanes 8 and 9 to 11 and 12). Quantification of the stabilization of ULBP1 in the presence of lysosomal protease inhibitors is shown in Figure 4, panel C. We have shown previously that U21 and class I MHC molecules are also stabilized in the presence of leupeptin and folimycin [32], [33]. To ensure that the effectiveness of our lysosomal protease inhibitors was comparable to inhibition observed in prior experiments, we immunoprecipitated U21 molecules from the same cells, in parallel (Figure 4, panel B, lanes 13–16, compare lanes 14 and 16). We note that the stabilization of ULBP1 resulting from U21 expression is not as dramatic as U21-mediated stabilization of class I MHC molecules. Nonetheless, these results, and the relocalization of ULBP1 to lysosomes in U21 expressing cells (Figure 3), suggest that U21 can also reroute ULBP1 to lysosomes for degradation.

**Figure 5:**
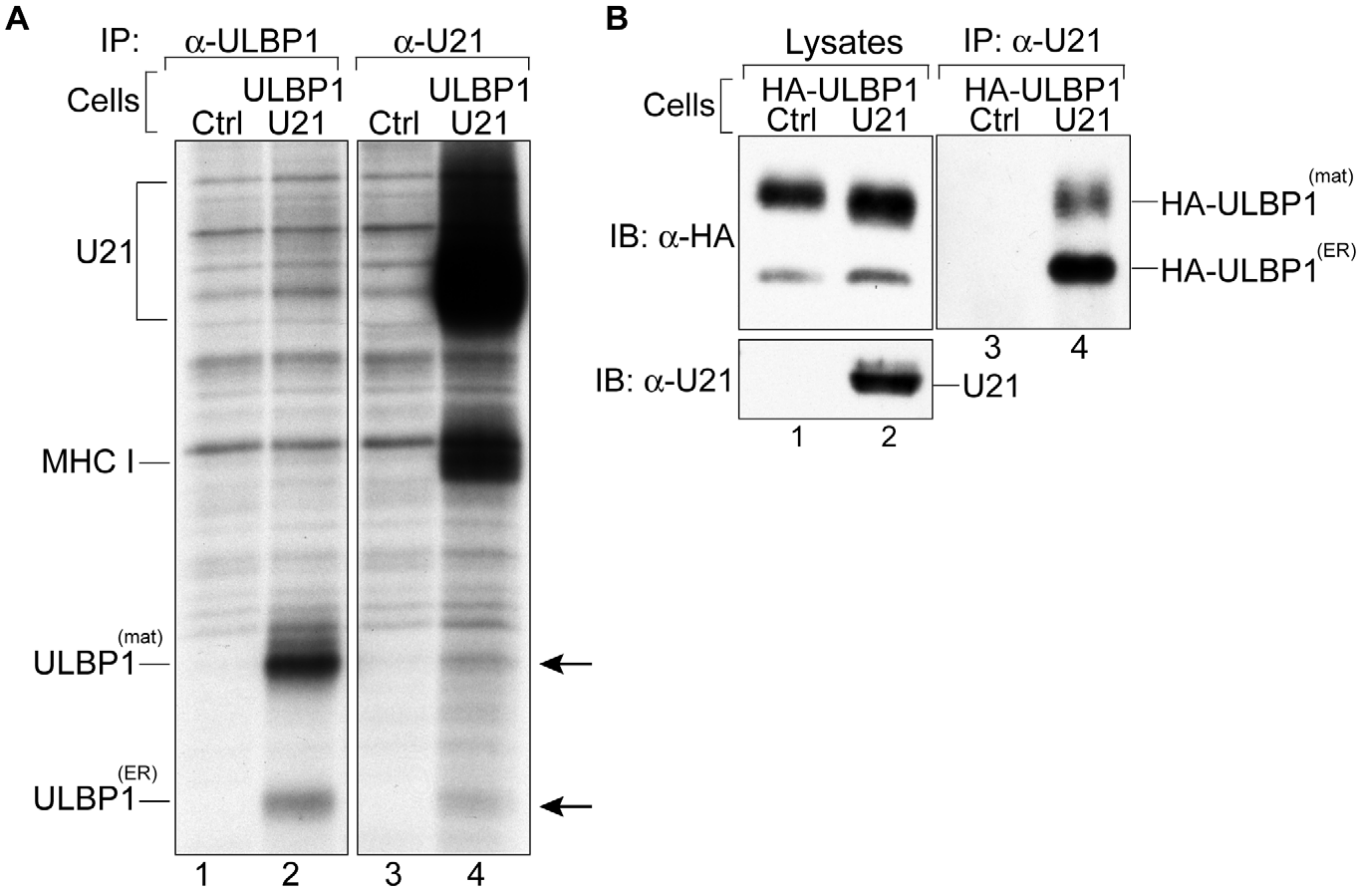
U21 interacts with ULBP1. (A) U373 and ULBP1-U21 cells were labeled for 2 hrs, and either U21 or ULBP1 (m295) was recovered from digitonin lysates. Migration positions of U21, class I MHC heavy chain, and ULBP1 are indicated. Arrows indicate potential coimmunoprecipitating polypeptides identical in size to ULBP1. (B) Cells, as indicated, were incubated in the presence of folimycin and leupeptin, and U21 was recovered from digitonin lysates. Lysates and immunoprecipitations were immunoblotted with anti-HA and anti-U21 antibodies. Migration positions of mature (HA-ULBP1^mat^) and immature (HA-ULBP1^ER^) HA-ULBP1 and U21 are noted.

When we recovered U21 from metabolically-labeled ULBP1-expressing cells, we observed labeled co-precipitating class I heavy chains, but we did not observe co-precipitating ULBP1 molecules (Figure 4, panel B, lanes 13–16). Likewise, when we recovered ULBP1, we observed no labeled coprecipitating U21 molecules (Figure 4, panel B, lanes 7–12, migration position of U21 is shown in lanes 13–16). We therefore performed coimmunoprecipitation experiments using lysis buffer containing digitonin rather than TritonX-100. Under these conditions, when we recovered U21, we observed coprecipitation of two polypeptides identical in size to ULBP1 (Figure 5, panel A, lane 4). In the reciprocal immunoprecipitation, however, when we recovered ULBP1 from digitonin lysates, we failed to observe co-precipitation of U21. It is possible that the anti-ULBP1 antibody precludes co-precipitation of U21 with ULBP1.

To confirm the identity of the polypeptides coimmunoprecipitating with U21 as ULBP1 molecules, we recovered U21 molecules from cells expressing HA-tagged ULBP1 (Figure S2), and immunoblotted the immunoprecipitates with an antibody directed against the HA epitope tag. Because steady-state levels of proteins destined for the lysosomal compartment can be difficult to detect, we sought to minimize the lysosomal degradation of U21 and HA-ULBP1 by preincubating the cells in the presence of lysosomal protease inhibitors. In lysates from digitonin-lysed HA-ULBP1-expressing cells, we observed both the ER-resident, non-GPI-linked form of HA-ULBP1, as well as the GPI-linked mature form, with the mature form predominant (Figure 5, panel B, lanes 1 and 2). When we immunoprecipitated U21 from HA-ULBP1 cells expressing U21, the HA antibody recognized both forms of HA-ULBP1 in the anti-U21 immunoprecipitation (Figure 5, panel B, lane 4). Thus, U21 associates with both the ER- and mature forms of ULBP1 molecules. These results suggest that U21 binds to ULBP1 in the ER and maintains its association with ULBP1 through the secretory pathway *en route* to lysosomes.

### U21 affects the half-life of MICB and alters its glycosylation

The localization of the MICA and MICB in U21-expressing cells is unusual; rather than punctate lysosomal localization, cells expressing U21 exhibit very little MIC labeling at all (Figure 2, panels N, O, Q, and R, asterisks). Given the structural similarity between class I MHC molecules, ULBPs, and the MIC proteins, we thought it likely that MICA and MICB would also be rerouted to lysosomes for degradation, and surmised that perhaps the half-life of the MICs in the lysosomal compartment might be too short to allow visualization of the MIC proteins in punctae.

We therefore examined the turnover of MICB in the presence of lysosomal protease inhibitors. Because MICA and MICB share 83% similarity, and because both proteins are similarly affected by U21, we chose to examine MICB rather than MICA, since in general, MICB-expressing cells exhibited more dim, yet visible, punctae than MICA-expressing cells (Figure 2, compare panels N and Q).

**Figure 6:**
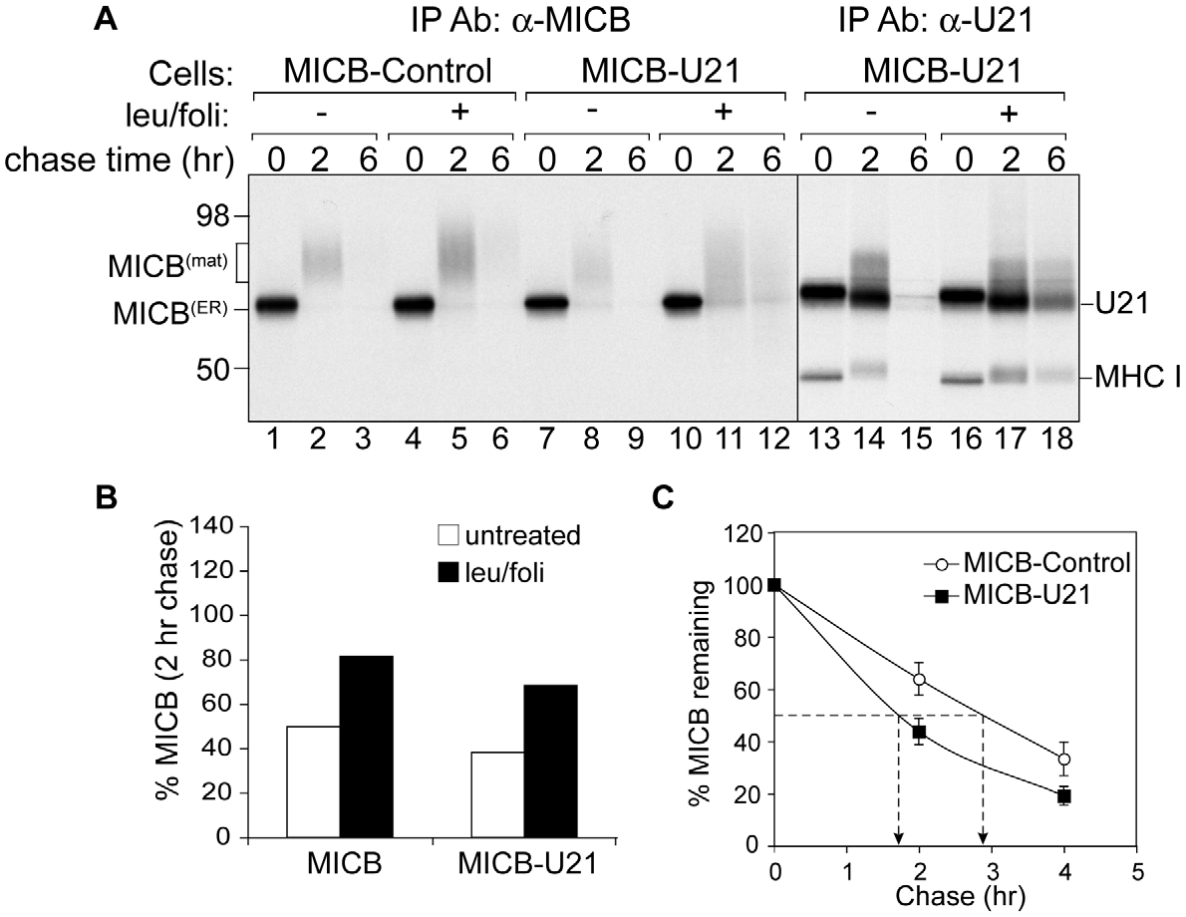
U21 expression destabilizes MICB. (A) MICB cells or MICB cells expressing U21 were pulse-labeled for 15 minutes and chased for 0, 2, or 6 hours. MICB (BMO2) and U21 were recovered from Triton X-100 lysates. Migration positions of MICB, U21, and class I MHC heavy chains are indicated, as are approximate molecular weight markers. Note that U21 and MICB migrate at nearly identical positions. (B) Quantification of (A) showing the percent MICB remaining after a 2 hr chase relative to the 0 hr chase point (n = 1). (C) The data represent the quantification of MICB stability in MICB or MICB-U21 cells from three independent experiments (n = 3). Error bars indicate the standard deviation.

In the absence of U21, after a 15 minute pulse-label, MICB migrated at a molecular mass of ∼60 kDa (Figure 6, panel A, lane 1). MICB became heterogeneously glycosylated (∼65–75 kDa) following a 2-hour chase period (Figure 6, panel A, lane 2), and no detectable MICB remained after 6 hours of chase (Figure 6, panel A, lane 3). Unlike class I MHC molecules, the cell surface expression of MICA and MICB is regulated by metalloproteinase cleavage, which results in shedding of the soluble extracellular domains from the cell surface, thus the half-life of MICB is likely the combined result of cleavage and release of soluble MICB by metalloproteinases [34]–[37] and of routine protein turnover [38]. Incubation of the MICB-expressing cells in lysosomal protease inhibitors resulted in slight stabilization of the MICB molecules (Figure 6, panel A, lanes 4–6, panel B, MICB), suggesting that some fraction of MICB is degraded in the lysosomal compartment.

In cells expressing U21, the turnover of MICB molecules was accelerated (Figure 6, panel A, lanes 7–9, and panel C), suggesting that, as for ULBP1 and class I MHC molecules, U21 might function to reroute MICB to the lysosomal compartment for degradation. However, lysosomal protease inhibitors stabilized MICB in U21 cells to approximately the same degree as in cells expressing MICB alone (Figure 6, panel A, lanes 10–12, and panel B), suggesting that U21 does not enhance the lysosomal degradation of MICB. To ensure that our lysosomal protease inhibitors were active, we recovered U21 from the same MICB-U21-expressing lysates, and found the lysosomal protease inhibitors were successful at stabilizing U21 and class I MHC molecules (Figure 6, panel A, lanes 13–18).

**Figure 7:**
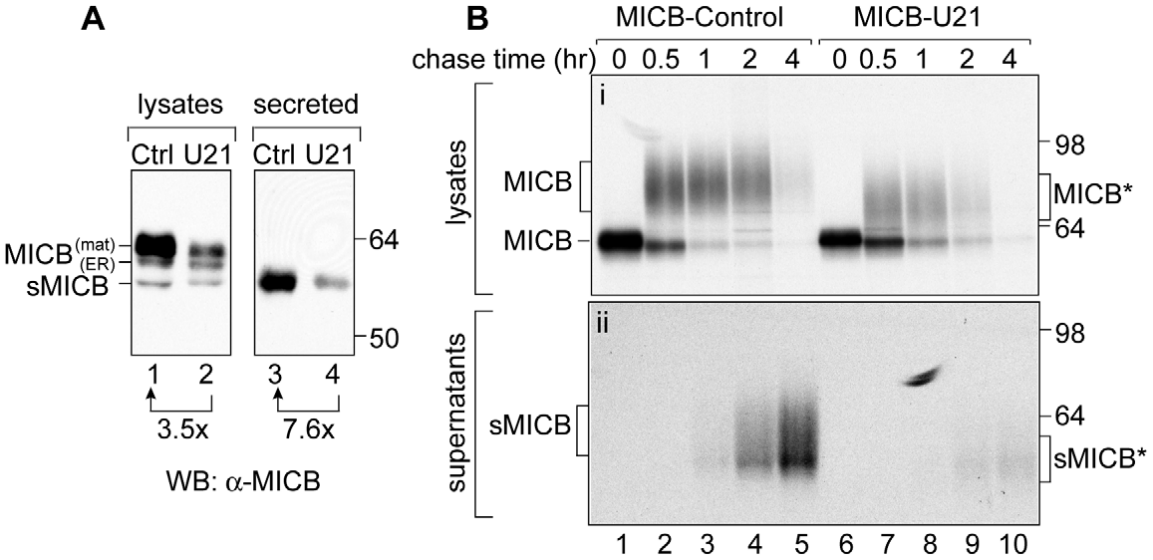
U21 expression reduces steady state levels of mature and secreted MICB. (A) Immunoblot analysis of lysates or supernatants from MICB or MICB-U21 cells. Lysates and concentrated supernatants were prepared from 4×10^5^ MICB or MICB-U21 cells. (B) MICB or MICB-U21 cells were pulse-labeled for 15 min and chased for 0, 0.5, 1, 2, and 4 hrs. MICB was recovered from Triton X-100 lysates (panel i) or cell supernatants (panel ii). Migration positions of MICB, soluble MICB (sMICB), and molecular weight markers are indicated. In U21-expressing cells, the faster-migrating mature (MICB*) and secreted (sMICB*) MICB are noted separately at the right.

Because the half-life of MICB was reduced in U21-expressing cells, we performed immunoblot analysis to examine the steady-state levels of MICB. In the absence of U21, we detected three polypeptides with an anti-MICB antibody (Figure 7, panel A, lane 1). The uppermost band corresponded in size to the mature form of MICB, and is the predominant form. The middle band corresponded in size to the immature ER form, and the lower band corresponded in size to soluble “shed” MICB. Expression of U21 resulted in a reduction in the steady-state level of MICB, primarily of the mature form (Figure 7, panel A, lane 2, top bands), suggesting that U21 expression results in the degradation of MICB after it has acquired Endo H-resistance, later in the secretory pathway.

We envisioned two possible explanations for the reduction in steady-state levels of MICB: U21 might enhance MICB degradation by either lysosomal or proteasomal proteases. However, neither lysosomal hydrolases nor proteasomal proteases appeared to participate appreciably in U21-mediated degradation of MICB (Figure 6, panels A and B, data not shown). Alternatively, we hypothesized that U21 might accelerate MICB's cleavage and release from the cell, resulting in the appearance of reduced steady-state levels of MICB. To examine the effect of U21 expression on the amount of secreted MICB in the supernatants, we examined the steady-state levels of MICB in supernatants of MICB- and MICB-U21-expressing cells. Rather than elevated levels of secreted MICB, we found a reduction in the amount of MICB present in the supernatant of U21-expressing cells, instead suggesting that U21 impairs the release of MICB (Figure 7, panel A, lanes 3 and 4).

To further evaluate the effect of U21 on MICB secretion, we performed a pulse-chase experiment over a shorter 4-hr chase period, recovering MICB from both lysates and supernatants after 0, 0.5, 1, 2, and 4 hours of chase (Figure 7B). The turnover of MICB was rapid; after 4 hours, there was very little labeled MICB recoverable from either control or U21-expressing cell lysates (Figure 7B, panel i, lanes 5 and 10). To examine shedding of MICB into the medium, we recovered MICB from the medium by immunoprecipitation and subjected it to SDS-PAGE. While we detected labeled secreted MICB in the medium of MICB-expressing cells (Figure 7B, panel ii, lanes 1–5), we recovered almost no MICB in the medium of U21-expressing MICB cells (Figure 7, panel ii, lanes 6–10), further suggesting that U21 impairs the shedding of MICB into the medium.

In cells expressing U21, the heterogeneously glycosylated MICB migrated slightly faster than MICB from control cells (Figure 7B, panel i, compare lanes 4 and 7), suggesting that U21 expression affected the trimming of either the MICB core polypeptide or its N-linked sugars. To determine whether U21 expression induced a reduction in the core polypeptide size of MICB, we digested MICB immunoprecipitates with PNGase:F (Figure S3). PNGase:F digestion resulted in polypeptides of identical mobility, suggesting that the core polypeptide remained unchanged, and that the U21-induced increase in MICB mobility must be the result of altered post-translational modifications to its N-linked glycans.

### U21 reduces sensitivity to NK cell lysis by down-regulating MICA and MICB

To examine whether the U21-induced downregulation of surface MICA and MICB could protect cells from NK recognition, we next performed NK cytotoxicity assays. NK-mediated cell lysis depends on the integrated response of NK cells to both inhibitory and activating ligands. Since U21 affects both class I MHC molecules (NK inhibitory ligands) and the MIC proteins (NK activating ligands), analysis of NK cytoxicity toward U21-expressing cells is complicated. To simplify the assessment of U21's effect upon MICA and MICB, we expressed U21 in the erythroleukemic cell line K562, which lack class I MHC molecules, thus any effect of U21 on NK cytotoxicity toward K562 cells should be independent of U21's ability to downregulate the surface expression of class I MHC molecules.

**Figure 8:**
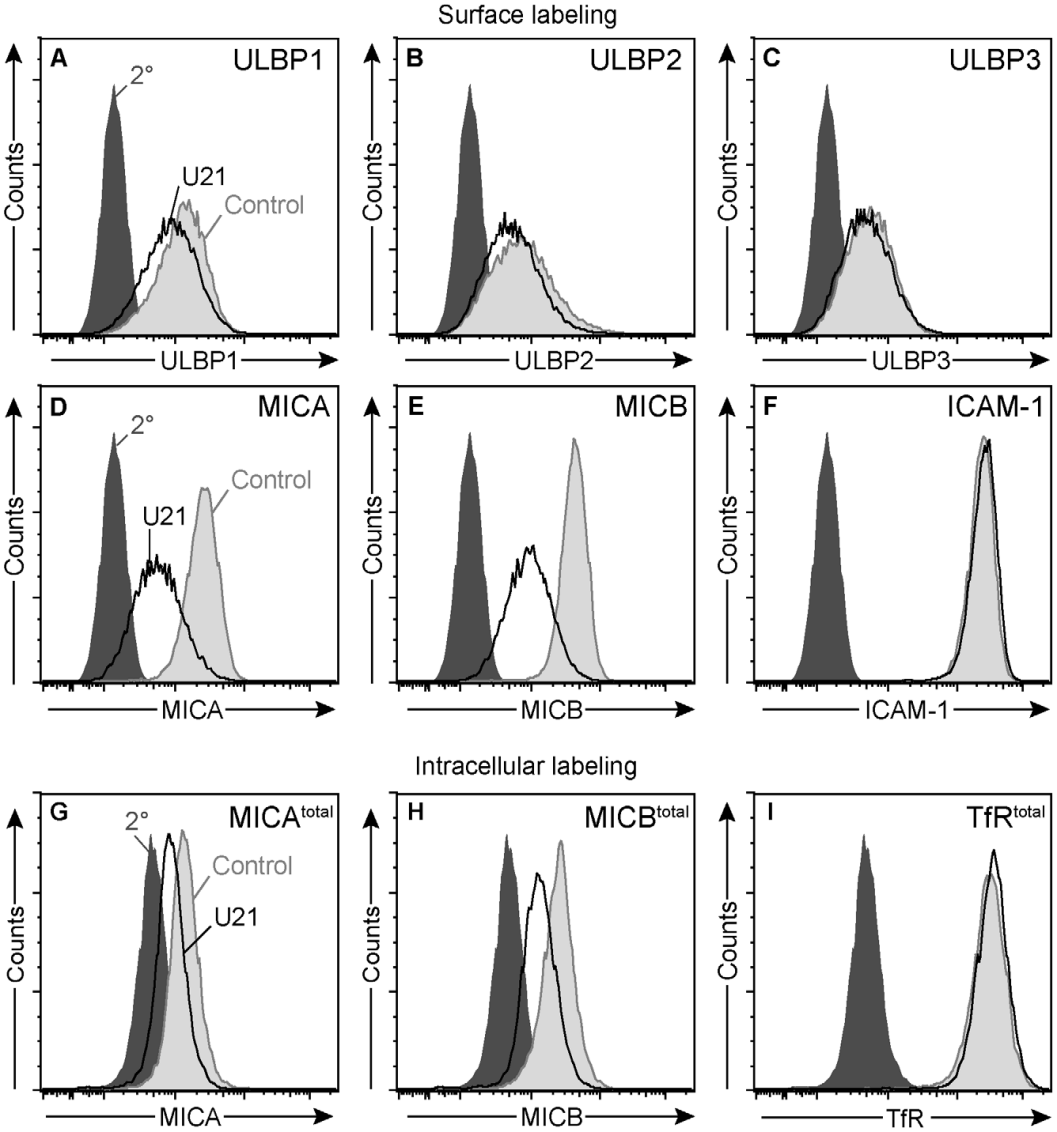
U21 down-regulates and destabilizes MICA and MICB in K562 cells. (A-F) Flow cytometric analysis of K562 cells expressing ZsGreen or U21-ZsGreen labeled with antibodies to (A) ULBP1 (m295), (B) ULBP2, (C) ULBP3, (D) MICA, and (E) MICB (m360), or (F) ICAM-1 followed by a PE-conjugated secondary antibody. Cells labeled with secondary antibody alone are indicated by the filled dark gray trace. Control ZsGreen-expressing cells are indicated by the filled light gray trace, and ZsGreen-U21 cells are indicated by the black trace. (G-I) Cells were fixed, permeabilized, and labeled with antibodies directed against (G) MICA, (H) MICB (m360), or (I) TfR. Cells labeled with secondary antibody alone are indicated by the filled dark gray trace. Control ZsGreen-expressing cells are indicated by the filled light gray trace, and ZsGreen-U21 cells are indicated by the black trace.

For these cytotoxicity assays, we generated a population of K562 cells stably expressing U21 (See Figure S4). When we examined the surface expression of the endogenous NKG2D ligands in the U21-expressing K562 cells, similar to our results in U373 cells, we observed a very slight decrease in the surface expression of endogenous ULBP1 in the U21-expressing K562 cells (Figure 8, panel A), and an even more prominent decrease in the surface expression of MICA and MICB (Figure 8, panels D and E). Surface levels of endogenous ULBP2 also appeared slightly reduced in the K562 cells, while the endogenous surface ULBP3 appeared to be unaffected (Figure 8, panels B and C), as was the surface expression of ICAM-1, an adhesion molecule critical for synapse formation between the NK and target cell (Figure 8, panel F) [39] (for review see [40]).

To further investigate whether the effects of U21 expression upon MICB were similar in both K562 and U373 cells, we also examined the half-life of MICB in the U21-expressing K562 cells. Because MICB expression in K562 cells is inherently low, we were unable to detect endogenous MICB by immunoblotting. We therefore performed intracellular labeling of MICA or MICB, comparing MIC expression levels using flow cytometry. Steady-state levels of MICA and MICB were reduced in K562 cells expressing U21 (Figure 8, panels G and H), suggesting that U21 expression can also result in the destabilization of MICA and MICB in K562 cells. In contrast, steady state levels of ICAM-1 (data not shown) or the transferrin receptor remained unaffected by U21 expression in these cells (Figure 8, panel I).

**Figure 9:**
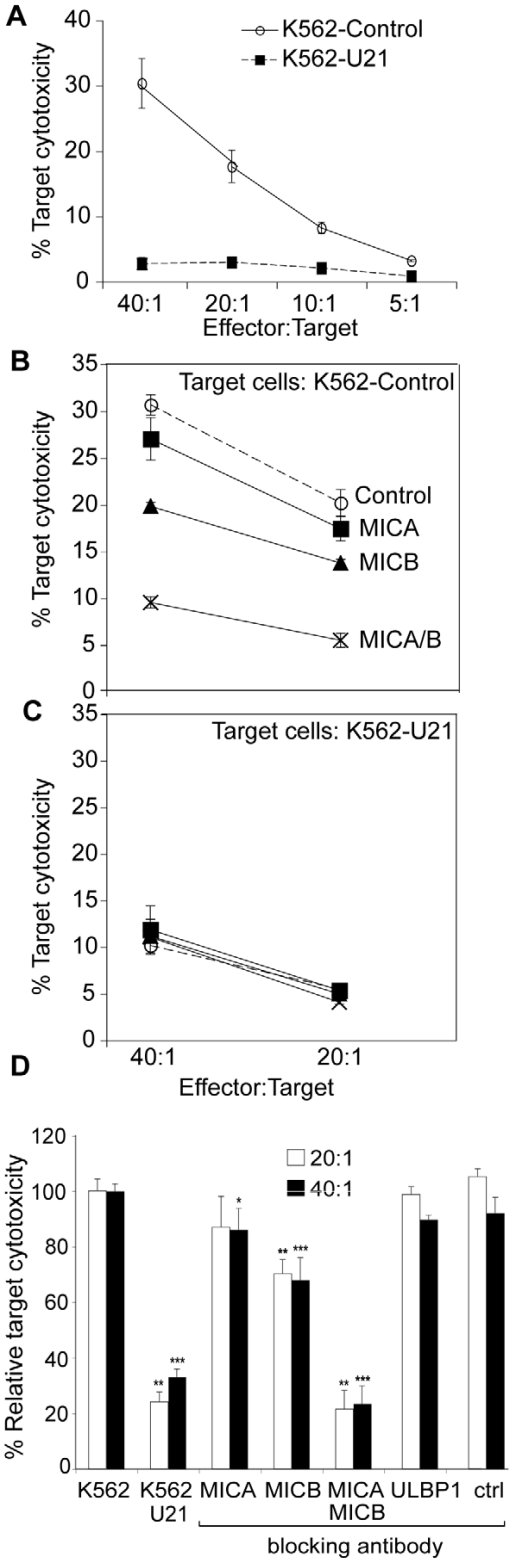
Expression of U21 in K562 cells reduces sensitivity to NKL-mediated cytotoxicity. (A) NK cytotoxicity assay. Target K562 cells expressing ZsGreen or U21-ZsGreen were incubated in the presence of NKL effector cells at the indicated E:T ratios. The graphs shown are single representative experiment done in triplicate and the error bars indicate the standard deviation between replicate samples (n = 1). (B-C) Target K562 cells expressing ZsGreen (B) or U21-ZsGreen (C) as indicated were incubated with 10 µg/ml IgG_1_ directed against, MICA, MICB (m360), or a combination of anti-MICA and anti-MICB, or no antibody (control) before addition of NKL effector cells at the indicated E:T ratios. The graphs shown are a single representative experiment performed in triplicate. Error bars indicate the standard deviation between the replicate samples (n = 1). (D) Compilation and statistical analysis of data from 3 independent experiments performed in triplicate (n = 3). The antibody blocking experiment was also performed once in triplicate (n = 1) using an antibody directed against ULBP1 (m295) or an IgG_1_ isotype control (m479). *pvalue<0.05, **pvalue<0.01, ***pvalue<0.001.

To evaluate the sensitivity of the U21-expressing K562 cells to NK cytotoxicity, we incubated the target K562 cells in the presence of effector NKL cells, and determined NK cell cytotoxicity using a flow cytometric assay. While control K562 cells were sensitive to NK cytotoxicity, cells expressing U21 were resistant to NK lysis (Figure 9, panel A). U21-expressing cells were also resistant to NK cytotoxicity from peripheral blood mononuclear NK cells (Figure S5).

To further define the mechanism of U21-mediated protection of K562 cells from NKL cytotoxicity, we performed NK cytotoxicity assays in the presence of blocking antibodies directed against MICA, MICB, and ULBP1. In control K562 cells, NK cytotoxicity was reduced by 15% in the presence of an antibody directed against MICA, by 25% in the presence of an antibody directed against MICB, and by 80% in the presence of both antibodies (Figure 9, panel B and D). This synergistic effect in the presence of blocking antibodies to both MICA and MICB suggest that the signal for NKL activation can be mediated through either ligand when one is unavailable, but when both are blocked, target cells are not able to activate the NKL cells. In contrast, pre-incubation of the K562 cells with an antibody directed against ULBP1 or an isotype control had no effect on cytotoxicity (Figure 9, panel D). Thus, the majority of NKL cytotoxicity between NKLs and K562 cells is likely mediated through engagement of MIC proteins on the surface of the target K562 cell with NKG2D on the NKL cells.

Blocking of both MICA and MICB on control K562 cells reduced the cytotoxicity to a level similar to that seen for U21-expressing K562 cells (Figure 9, panel C). Unlike control K562 cells, the cytotoxicity toward U21-expressing K562 cells was not further reduced by pre-incubation with antibodies directed against MICA or MICB (Figure 9, panel C). Since NKL-mediated cytotoxicity of K562 cells is largely mediated through MICA and MICB, the ability of U21 to reduce the surface expression of MICA and MICB likely contributes significantly to the mechanism by which U21 protects K562 cells from NK cytotoxicity.

If U21 can bind to and reroute ULBP1 molecules to the lysosomal compartment in U373 cells overexpressing ULBP1, why are endogenous ULBP1 surface levels in K562 cells only minimally reduced by U21, and why does ULBP1 seem to play no appreciable role in NK cytotoxicity toward K562 target cells? One possibility to explain the lack of NKL-mediated cytotoxicity through ULBP1 in the K562 cells may be the relative level of surface-expressed ULBP1 and its affinity for NKG2D. To explore the possibility that the surface expression of ULBP1 is insufficient to provide ligand for NKG2D, we overexpressed the ULBP1 activating ligand in K562 target cells. When ULBP1 was overexpressed, we observed an increase in NK cytotoxicity toward ULBP1 expressing cells (data not shown). We conclude that the constitutively-expressed ULBP1 on the surface of K562 cells may not be sufficient to induce NKL-mediated killing through engagement of NKG2D. ULBP1-expressing U373 cells, on the other hand, express abundant ULBP1, yet U21 has little effect upon the surface expression of ULBP1 in these cells. We therefore also think it likely that the affinity of U21 is lower for ULBP1 than for MICA and MICB. U21 does not act upon ULBP1 as effectively as it acts upon the MICs and class I MHC molecules.

## Discussion

HHV-7 U21 has long been known to bind to and reroute class I MHC molecules to the lysosomal compartment, likely providing HHV-7 a means of escaping detection by CTLs (2001). We have now demonstrated that U21 can reduce NK-mediated cytoxicity as well, by affecting surface expression of the NKG2D ligands MICA and MICB.

The MIC proteins are structurally similar to class I MHC molecules, and U21 expression resulted in the reduced surface expression of both the MICs and class I MHC molecules, thus we surmised that U21 acts upon the MICs and class I MHC molecules in a similar manner. However, we were unable to demonstrate association between U21 and the MIC molecules. Moreover, the MICs do not seem to be intensely localized to lysosomes in U21-expressing cells, nor is their stabilization by lysosomal protease inhibitors increased upon U21 expression. Why this difference? It is possible that U21 acts differently upon MICs than upon class I MHC molecules. Alternatively, the reagents available to detect the MIC molecules may not be sufficient to follow the MIC molecules as they traffic to lysosomes. The MIC family of NKG2D ligands is highly regulated. MIC expression at the transcriptional level is upregulated in response to stressors such DNA damage, autoimmunity, and infection (For review, see [41]). In cells that express MIC proteins, the trafficking of the MICs is also complex; MIC expression at the cell surface is regulated both by internalization and recycling, as well as by shedding of the MIC molecules into the medium [34], [37], [38]. Perhaps because of the relative complexity of the trafficking of MIC molecules, U21's molecular impact upon the MIC molecules is less straightforward.

Unlike class I molecules, even in the absence of U21, MICB is partially stabilized by lysosomal protease inhibitors. If U21 functioned to enhance the routing of MICB to lysosomes, we would have expected to observe enhanced stabilization of MICB by lysosomal protease inhibitors, but we did not (Figure 6). We therefore reasoned that if the MICB that disappears during the chase period is not degraded in lysosomes, then it must be shed or released from the cell. In control MICB-expressing cells, this indeed appears to be the case. In cells expressing U21, however, MICB appears to be neither degraded in lysosomes nor released into the supernatant (Figure 7). We can think of two possibilities to explain these observations: either MICB is shed, but the MICB shed from U21-expressing cells is less stable, explaining why we are unable to recover it from the medium, or, perhaps MICB interacts with U21 within the cell, rendering it undetectable by MICB antiserum. We also found that U21 expression resulted in altered glycosylation of MICB, thus perhaps this altered glycosylation of MICB may affect its stability upon release into the extracellular environment. Alternatively, it is possible that interaction of U21 with MICB precludes its shedding by the metalloproteases that cleave it [37].

Several similar trafficking studies examining the mechanism for HCMV immunoevasin UL16-mediated effect upon MICB have been performed [15], [42]. Like U21, UL16 localizes to the ER and Golgi region [15], [42], reduces the cell-surface expression of MICB [15], and impairs the release of soluble MICB into the medium [43]. But, as for U21, the complexity of MIC regulation and trafficking has impeded a clear understanding of how UL16 affects MICB trafficking [15], [42], [43].

The experiments described herein also illuminate some of the mechanistic and structural aspects of the interaction between U21 and class I MHC molecules: the discovery that U21 binds to the class I MHC-like ULBP1 contributes to our understanding of U21's interaction with class I MHC and class I-like molecules. U21 can associate with a wide range of class I MHC molecules, including HLA-A, -B, -C, -E, -G, and even murine class I MHC molecules [28], [32]. The greatest degree of conservation among all of these class I molecules exists within the α3 domain of these proteins, thus we originally hypothesized that the α3 domain was an important structural feature for association of U21 with these molecules [28] (see schematic, Figure 1, panel A). Interestingly, ULBP1 possesses α1 and α2 domains, but lacks an α3 domain. The structural similarities between class I MHC molecules and ULBP1 would therefore suggest that the binding of U21 to class I molecules involves the α1 and/or α2 domains.

U21 expression also results in the relocalization of ULBP1 to lysosomes. However, when U21 is expressed in a cell line that offers a choice of substrates - ULBP1 or class I MHC molecules - the choice is clear: U21 has a more striking affect upon class I MHC molecules than upon ULBP1, suggesting that U21 may possess greater affinity for class I MHC molecules than for ULBP1. It is important to note, however, that our experiments were performed in cells exogenously overexpressing ULBP1, or in K562 cells, which express low constitutive levels of ULBP1. In the context of an HHV-7 infection, it is possible that the relative expression of ULBP1 and class I MHC molecules may be such that U21 can act effectively upon ULBP1 molecules. However, when comparing the effect of U21 upon all of the NKG2D ligands, it is clear that U21 expression causes a much greater reduction in the cell surface expression of the MIC molecules than of the ULBPs, suggesting that U21's primary effect may be upon the MIC molecules.

U21's true utility as an immunoevasin during HHV-7 infection is, as yet, difficult to assess, because a bacterial artificial chromosomal system to facilitate genetic manipulation of the viral genome has not yet been established, and HHV-7 is a human herpesvirus for which there is no animal model. Additionally, although HHV-7 is known to infect T cells, the site of infection where immune escape is most critical for the virus is not certain; HHV-7 is shed in the saliva of healthy individuals, thus salivary glands may be a site of persistent infection where evasion of NK cytotoxicity is essential. It is also possible that, as for closely-related rhesus CMV, immunoevasin involvement in the escape of immune detection is important not during primary infection, but during superinfection with other strains [44].

HHV-7 U21 can bind to and affect the surface expression of many different HLA class I alleles, including the NK-inhibitory ligands HLA-C and HLA-E, downregulation of which, in principle, should render the cell susceptible to NK attack. We had therefore hypothesized that HHV-7 must encode other means of NK cell evasion [28]. We now demonstrate that HHV-7 U21 also reduces the surface expression of the NK activating ligands MICA and MICB, thereby preventing NK cytoxicity toward U21-expressing cells. Thus HHV-7, through a single viral protein, encodes a means to escape both CTL and NK cell detection. Interestingly, like U21, at least two other viral proteins can affect both CTL and NK cell recognition. The murine CMV m152 gene product (gp40) causes retention of both class I MHC molecules and NKG2D-ligands in the endoplasmic reticulum-Golgi intermediate compartment [21], [22], [45]. Additionally, unlike MCMV, which encodes multiple means of affecting both CTL and NK detection, Adenovirus is known to encode only one polypeptide, E3/19K, which can influence both CTL and NK recognition. E3/19K binds to class I MHC molecules and to MICA and MICB molecules and retains them in the ER [24], [46], [47]. Thus, MCMV gp40, Adenovirus E3/19K, and HHV-7 U21 all recognize multiple structurally-similar class I MHC and class I MHC-like molecules and may possess dual function during viral infection.

## Materials and Methods

### Cell lines

U373 and HEK293T were cultured in Dulbecco's modified Eagle medium (DMEM) supplemented with 5% newborn calf serum (NCS) and 5% fetal bovine serum (FBS) in the presence of puromycin (375 ng/ml) (Sigma-Aldrich, St. Louis, MO) or geneticin (G418) (500 ng/ml)(Invitrogen, Carlsbad, CA), as needed. K562 cells were cultured in RPMI supplemented with 10% FBS. NKL cells (generously provided by Dr. M. J. Robertson, Indiana University) were cultured in RPMI supplemented with 10% heat-inactivated FBS, 1 mM sodium pyruvate and 50–100 U/ml IL-2. U373 cells stably expressing the NKG2D ligands ULBP1, ULBP2, or ULBP3, or MICA or MICB were generated by retroviral transduction using the vector, pLNCX (Clontech, Mountain View, CA). In some cases, clones were isolated to generate cell lines with homogeneous expression of the NK activating ligand. These cell lines were then transduced with a lentiviral vector, pHAGE-puro-MCS (PPM)-U21, in which U21 was expressed under the control of a CMV promoter, and an IRES-driven puromycin N-acetyl transferase gene (*Pac*) allowed for puromycin selection [48]. K562 cells stably expressing U21 were generated by lentiviral transduction using a vector identical to PPM but containing the gene for Zs-Green instead of the *Pac* gene (PMG) [48]. The multiple cloning site (MCS) and puromycin cassette were modifications made to the pHAGE vectors in our laboratory, where we excised the gene for ZsGreen and replaced it with the puro cassette. The MCS was inserted in place of the gene for Ds-Red.

### Antibodies

Monoclonal antibodies to ULBP1 (m295, IgG_1_), ULBP2 (m311, IgG_1_), ULBP3 (m551, IgG_1_) ULBP4 (m479, IgG_1_), MICA (m673, IgG_1_), and MICB (m360, IgG_1_) were generously provided by Amgen (Thousand Oaks, CA). Affinity purified goat polyclonal antibodies directed against ULBP1 (AF1380) and MICB (AF1599) were purchased from R&D systems (Minneapolis, MN). BMO2, a monoclonal antibody directed against MICB, was purchased from Axxora (San Diego, CA). The anti-lamp2 monoclonal antibody H4B4 was generously provided by Dr. T. August (Johns Hopkins Medical School, Baltimore, MD). The transferrin receptor (TfR) monoclonal antibody, (anti-CD71) (clone H68.4) was purchased from Zymed Laboratories (San Francisco, CA). Monoclonal anti-glyceraldehyde-3-phosphate dehydrogenase (GAPDH) was purchased from Imgenex (San Diego, CA). The intercellular adhesion molecule-1 (ICAM) monoclonal antibody (anti-CD54) was purchased from BD Biosciences. W6/32 is a monoclonal antibody that recognizes properly-folded class I MHC molecules [49]. HC10 is a monoclonal antibody that recognizes free class I MHC heavy chains [50]. Fluorescein isothiocyanate (FITC)-conjugated W6/32 was purchased from eBiosciences (San Diego, CA). HA.11 is a monoclonal antibody directed against hemagglutinin (HA), and was purchased from Covance (Princeton, NJ). A polyclonal antibody (MCW50) directed against the cytoplasmic tail of U21 was generated in our laboratory [31].

### Construction of retroviral and lentiviral vectors

ULBP1–3, MICA, and MICB were amplified from plasmids provided by Dr. D. Cosman and cloned in to LNCX using the primers below. Constructs were verified by DNA sequencing. U21 was subcloned from PPM-U21 into PMG using XhoI and BamHI.

ULBP1:5'-ATACTCGAGGCCACC**ATGGCAGCGGCC     GCCAG**

3'-GTCAGGCTT **TCATCTGCCAGCTAGAAT    GAAG**

ULBP2:5'-AGTCTCGAGGCCACC**ATGGCAGCAGCC     GCCGC**

3'-GTCAAGCTT **TCAGATGCCAGGGAGGATG**

ULBP3:  5'-AGTCTCGAGGCCACC**ATGGAGACAG**

3'- GTCAAGCTT **TCAGATGCCAGGGAGGATG**

MICA: 5'-AGTCTCGAGGCCACC**ATGGGGCTGGGC     CCGG**

3'-GTCAAGCTT **CTAGGCGCCCTCAGTGG**

MICB: 5'-AGTCTCGAGGCCACC**ATGGGGCTGGGC     CGGG**

3'-GTCAAGCTT **CTAGGTGCCCTCAGTGG**

### Retroviral and lentiviral transductions

Packaging, envelope, and vector plasmids were cotransfected into HEK293T cells using TransIT-293 (Mirus Bio, Madison, WI). Viral supernatants were harvested at 48–72 hrs, filtered and either used to infect desired cell lines directly (retroviral transductions) or concentrated prior to infections (lentiviral transductions). Lentiviruses were concentrated by ultracentrifugation for 3 hrs at 4°C at 50,000xg. K562 cells were infected twice by spinoculation (1000 X g for 2 hr at 30°C) with concentrated lentiviruses. For G418- and puromycin-resistant constructs, cells were cultured in selection medium for at least 10 days.

### Immunofluorescence microscopy

Cells grown on glass coverslips were washed with PBS, fixed with 4% paraformaldehyde in PBS, permeabilized with 0.5% saponin in PBS and 3% BSA, incubated with primary antibodies, washed and incubated with Alexa488- or 594-conjugated secondary antibodies (Invitrogen). Colocalization studies (Figure 3) were performed using a Zenon Alexafluor 488 kit (Invitrogen) to label anti-lamp2 and the images were deconvoluted using Auto Quant 3D deconvolution software (Media Cybernetics, Bethesda, MD).

### Flow cytometry

Adherent cells were detached with trypsin or 5 mM EDTA in PBS prior to labeling. Cells were washed in ice-cold PBS, and incubated with primary antibodies in 1% bovine serum albumin/phosphate buffered saline (BSA/PBS) for 30 min on ice. For nonconjugated primary antibodies the cells were then washed with 1% BSA/PBS and incubated with Goat F(ab)_2_ Anti-Mouse IgG (H+L) Phycoerythrin (PE)(R&D Systems, Minneapolis, MN). For intracellular staining, cells were fixed with 1% paraformaldehyde (PFA) and permeabilized with 0.1% saponin prior to staining. Flow cytometry was performed on either a FACSCalibur or FACSAria III (BD Biosciences), or Guava Easycyte mini (Millipore, Billerica, MA) and the data was analyzed using FlowJo software (Treestar, Ashland, OR).

### Pulse-chase experiments

Cells were detached with trypsin and incubated in methionine- and cysteine-free DMEM (Invitrogen) supplemented with 2%FBS for 30 min at 37°C (starve). The cells were labeled with 700 µCi/ml of [35S]-Express label (1100 Ci/mmol; PerkinElmer, Boston, MA) at 37°C and chased with complete DMEM supplemented with 1 mM non-radioactive methionine and cysteine for indicated times at 37°C. Cells were washed with PBS then lysed in Triton X-100 lysis buffer (10 mM Tris-HCl [pH 7.4], 150 mM NaCl, 1%Triton X-100, 0.1 mM phenylmethylsufonyl fluoride (PMSF), and 5 mM N-ethyl-maleimide (NEM)) or digitonin lysis buffer (1% digitonin, 150 mM NaCl, 50 mM Tris-HCl [pH 7.4], 5 mM NEM, 0.1 mM PMSF) for 5 min at 37°C to solubilize lipid rafts, followed by rocking for 15 min at 4°C. Lysates were centrifuged for 10 min at 16,000 X g at 4°C to pellet nuclei and debris. Clarified lysates were incubated overnight at 4°C with designated antibodies and Protein A agarose (Repligen Corporation, Waltham, MA) or protein G agarose (Invitrogen). Immunoprecipitates were washed four times with Triton X-100 wash buffer (10 mM Tris 7.4, 150 mM NaCl, 1%Triton X-100) or digitonin wash buffer (0.1% digitonin, 150 mM NaCl, 50 mM Tris pH 7.4), and subjected to SDS-PAGE gel electrophoresis. When indicated, lysosomal inhibitors leupeptin (Sigma) and folimycin (EMD, San Diego, CA) were added at 200 µM and 20 nM, respectively, during the starve, pulse, and chase.

### Quantification of pulse-chase analysis

Quantification was performed from phosphorimages generated on a Storm 820 (GE Healthcare, Piscataway, NJ) using ImageQuantTL software. In general, when measuring the half-life of a protein by pulse-chase analysis, the amount of protein recovered at each time point is calculated as a percent of the protein recovered immediately following the pulse. We used this method to quantify the stability of MICB (Figure 8 panels B and C). However, since immunoprecipitation of immature form of ULBP1 immediately following the pulse is inefficient, we felt it appropriate to normalize to the amount of mature ULBP1 recovered after the 2 hr chase (Figure 5 panel B). All values are also corrected for background. Calculations were performed as follows: For Figure 5, the % ULBP1 =  (ULBP1^6hr^ - bkgd)/(ULBP1^2hr^ - bkgd)*100. For Figure 8, panel B %MICB =  (MICB^2hr^ - bkgd)/(MICB^0hr^ - bkgd)*100. For Figure 8, panel C all points are normalized to the 0 hr chase point as in B). For Figure 9, panel C, the %MICB secreted at each time point  =  (MICB^supernatant^ - bkgd)/(MICB^lysate 0hr^ - bkgd)*100.

### Immunoprecipitation and immunoblotting

Total cell lysates were prepared in 1% Triton X-100 lysis buffer supplemented with 50 U/ml benzonase (Sigma), followed by the addition of an equal volume of 2% SDS and 100 mM Tris-HCl [pH 7.4] and continued rocking at room temperature for 15 min. Lysates were normalized to total protein concentration as determined by BCA assay (Pierce, Rockford, IL). For immunoblot analysis of secreted MICB, supernatants were collected and concentrated 10 fold using a micron 30 filter (Millipore, Billerica, MA). For immunoprecipitation-immunoblot experiments, cells were treated with 200 µM leupeptin and 20 nM folimycin for 14 hours then lysed in digitonin lysis buffer. Immunoprecipitions were performed, washed 4 times with digitonin wash buffer, and eluted with Laemmli buffer. Lysates and immunoprecipitates were resolved by SDS-PAGE electrophoresis, transferred to BA-85 nitrocellulose membrane (Whatman, Florham Park, NJ) and probed with designated primary antibodies followed by an appropriate HRP conjugated secondary antibody (BioRad, Hercules, CA). Bands were visualized using SuperSignal reagent (Pierce) and quantified with an Alpha Imager (AlphaInnotech, San Leandro, CA).

### NK cytotoxicity assays

Target cells (2.5×10^4^) cultured in the presence of IL-2 (50 U/ml) were mixed with various ratios of NKL cells in V-bottom 96 well plates. When indicated, human PBMCs were isolated from blood on Ficoll-Paque (GE Healthcare) according to manufacturer's instruction and used in place of NKL cells. Plates were centrifuged for 5 min at 125 X *g* to pellet cells, and incubated for 3 hr at 37°C. Cells were then stained with 4 µg/ml 7-aminoactinomycin D (7-AAD)(Sigma) for 5 min and analyzed by flow cytometry. The percentage of target cell death was calculated as the %7-AAD-positive target cells (ZsGreen-positive) at each effector:target ratio minus the %7-AAD-positive target cells in the absence of NKL cells. When indicated, target cells were incubated with 10 µg/ml blocking antibodies for 15 minutes at 37°C prior to the addition of NKL cells. All assays were performed in triplicate and, unless noted, the experiments shown were the average of three independent experiments.

## Supporting Information

Figure S1 **Cells with higher U21 expression display more dramatic relocalization of ULBP1 to lysosomes.** (A and B) U373 cells expressing ULBP1 and U21 were double-labeled with antibodies directed against ULBP1 (m295) and U21. The arrows indicate a cell expressing a high apparent level of U21, the arrowheads indicate a cell expressing a lower apparent level of U21, and the asterisks indicate a cell that does not appear to express U21. (C) U373-ULBP1 cells were infected with increasing amounts of U21 retrovirus (lanes 2-7) and selected in puromycin to generate stable cell lines. U373 cells and cells expressing ULBP1 alone are shown in lanes 1 and 8, for comparison. Cell lysates (20 µg) from each cell line were immunoblotted with antibodies directed against U21, class I MHC heavy chain (HC10), ULBP1 (AF1380), or TfR. as indicated. As more U21 is expressed, the steady-state levels of both class I heavy chains (αMHC-I) and ULBP1 (αULBP1) are reduced. (D) As more U21 is expressed, relocalization of ULBP1 becomes more evident. Relocalization (appearance of puncta as depicted in panel B (arrowhead and arrow)) was quantified in each of the six cell lines depicted in panel C, lanes 2-7. 1000 cells were counted and scored for the presence of ULBP1-positive punctae (n = 1). Bars reflect the percentage of cells containing ULBP1-positive punctae from each cell line.
(TIF)

Figure S2 **U21 expression induces relocalization of HA-tagged ULBP1.** Immunofluorescent detection of ULBP1 in U373 cells expressing HA-ULBP1 (A) or HA-ULBP1 and U21 (B). Cells were labeled with an antibody directed against ULBP1 (m295) followed by an Alexa 488-conjugated secondary antibody, as indicated.
(TIF)

Figure S3 **Core, deglycosylated MICB migrates identically in MICB- and in MICB-U21-expressing cells.** U373 cells expressing MICB or MICB and U21 were pulse-labeled for 15 minutes and chased for 0, 2, or 6 hours. MICB (BMO2) was recovered from Triton X-100 lysates and treated with either Endo H or PNGase:F. Migration positions of EndoH sensitive MICB (MICB(s)), PNGase:F resistant MICB (MICB(r)), and deglycosylated MICB (deglycos) are indicated, as are approximate molecular weight markers (right).
(TIF)

Figure S4 **Expression of U21 in K562 cells.** (A) Flow cytometric analysis of ZsGreen from K562 cells (dark gray solid), K562 cells expressing Zs-Green (light gray solid), or K562 cells expressing U21-IRES-ZsGreen (black line). (B) Cell lysates (30 µg) from ZsGreen- or U21-ZsGreen-expressing K562 cells were immunoblotted with an antibody directed against U21. A cross-reactive polypeptide recognized by the polyclonal U21 antibody serves as a loading control and is denoted with an asterisk.
(TIF)

Figure S5 **Expression of U21 in K562 cells reduces sensitivity to cytotoxicity mediated by human NK cells.** Target K562 cells expressing ZsGreen or U21-ZsGreen were incubated in the presence of freshly isolated human peripheral blood mononuclear NK effector cells at the indicated E:T ratios. The graphs shown are single representative experiment performed in triplicate, and the error bars indicate the standard deviation between replicate samples (n = 1).
(TIF)
