# Supplementary figures and images for: The Human Herpesvirus-7 (HHV-7) U21 Immunoevasin Subverts NK-Mediated Cytoxicity through Modulation of MICA and MICB
Source: PLoS Pathog. 2011 Nov 10;7(11):e1002362. doi: 10.1371/journal.ppat.1002362 (PMC3213103; doi:10.1371/journal.ppat.1002362)

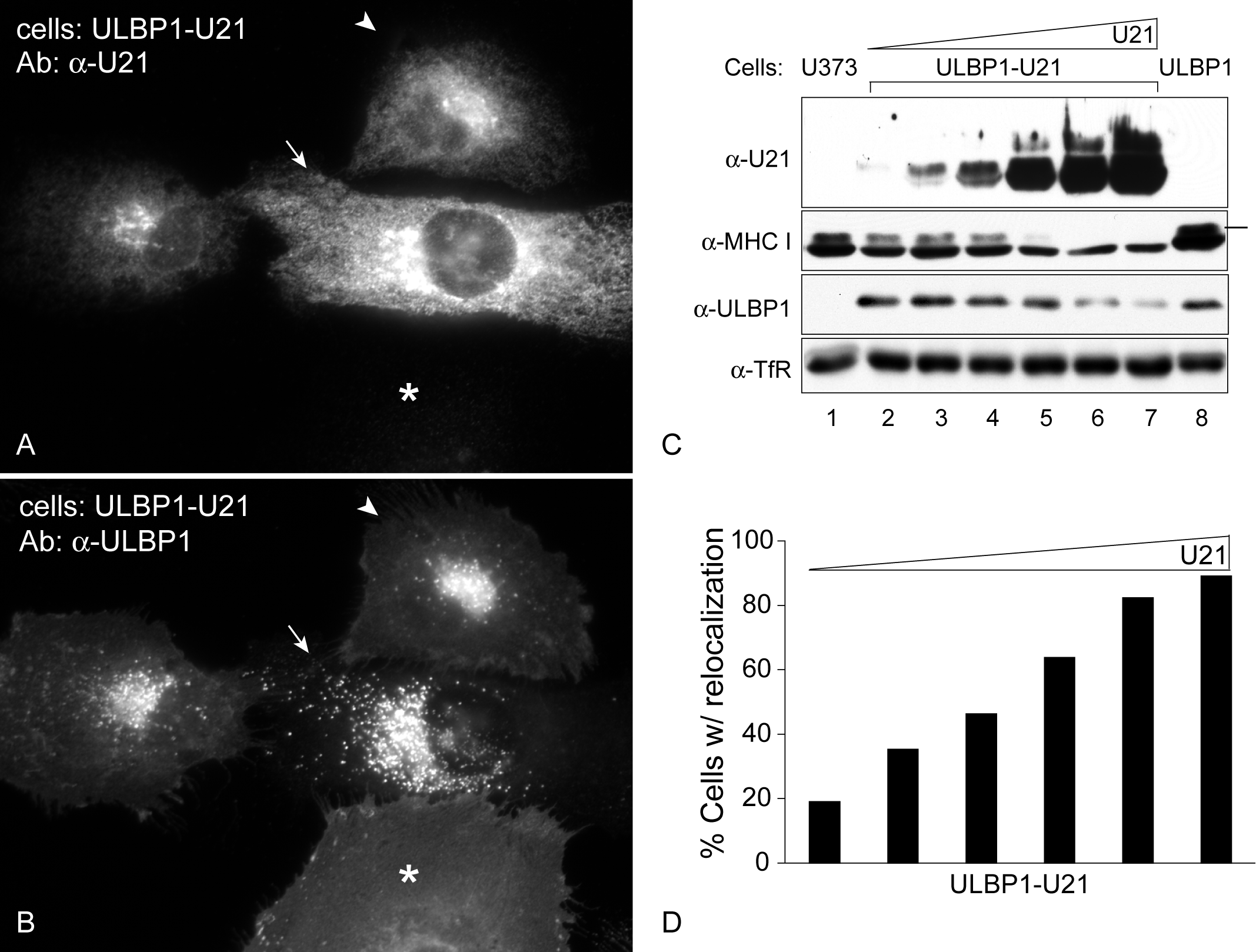

Supplement: Figure S1 — Cells with higher U21 expression display more dramatic relocalization of ULBP1 to lysosomes. (A and B) U373 cells expressing ULBP1 and U21 were double-labeled with antibodies directed against ULBP1 (m295) and U21. The arrows indicate a cell expressing a high apparent level of U21, the arrowheads indicate a cell expressing a lower apparent level of U21, and the asterisks indicate a cell that does not appear to express U21. (C) U373-ULBP1 cells were infected with increasing amounts of U21 retrovirus (lanes 2-7) and selected in puromycin to generate stable cell lines. U373 cells and cells expressing ULBP1 alone are shown in lanes 1 and 8, for comparison. Cell lysates (20 µg) from each cell line were immunoblotted with antibodies directed against U21, class I MHC heavy chain (HC10), ULBP1 (AF1380), or TfR. as indicated. As more U21 is expressed, the steady-state levels of both class I heavy chains (αMHC-I) and ULBP1 (αULBP1) are reduced. (D) As more U21 is expressed, relocalization of ULBP1 becomes more evident. Relocalization (appearance of puncta as depicted in panel B (arrowhead and arrow)) was quantified in each of the six cell lines depicted in panel C, lanes 2-7. 1000 cells were counted and scored for the presence of ULBP1-positive punctae (n = 1). Bars reflect the percentage of cells containing ULBP1-positive punctae from each cell line. (TIF) [file ppat.1002362.s001.tif]

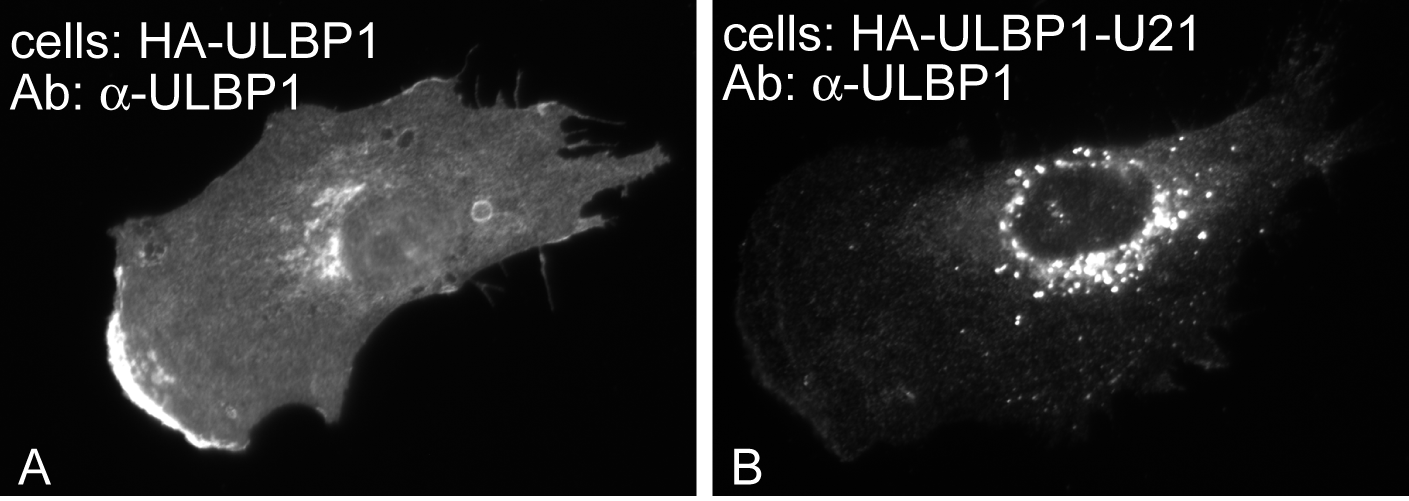

Supplement: Figure S2 — U21 expression induces relocalization of HA-tagged ULBP1. Immunofluorescent detection of ULBP1 in U373 cells expressing HA-ULBP1 (A) or HA-ULBP1 and U21 (B). Cells were labeled with an antibody directed against ULBP1 (m295) followed by an Alexa 488-conjugated secondary antibody, as indicated. (TIF) [file ppat.1002362.s002.tif]

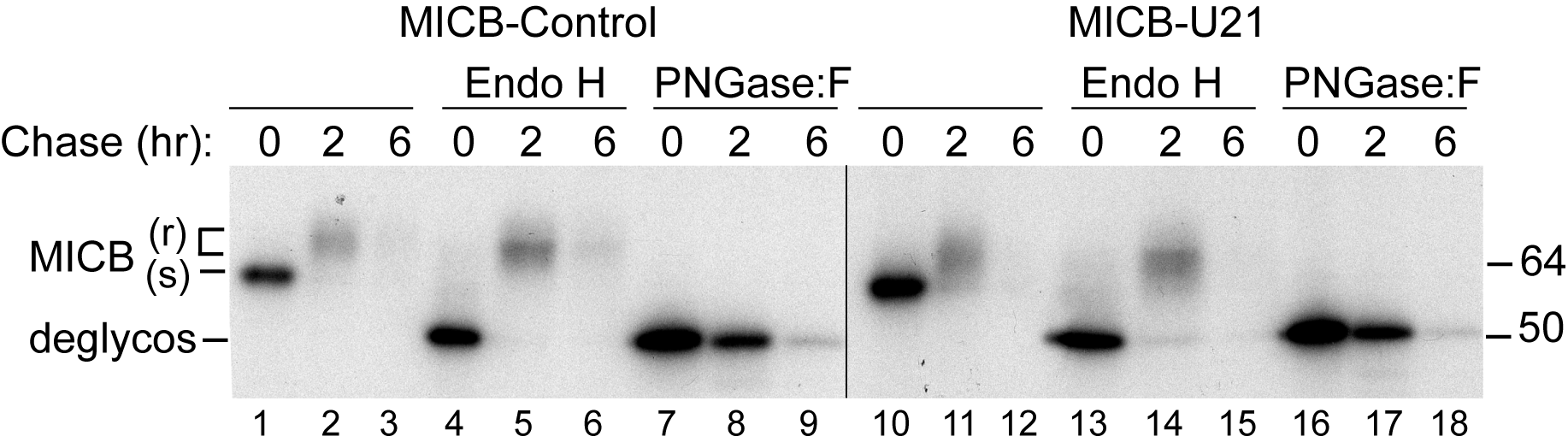

Supplement: Figure S3 — Core, deglycosylated MICB migrates identically in MICB- and in MICB-U21-expressing cells. U373 cells expressing MICB or MICB and U21 were pulse-labeled for 15 minutes and chased for 0, 2, or 6 hours. MICB (BMO2) was recovered from Triton X-100 lysates and treated with either Endo H or PNGase:F. Migration positions of EndoH sensitive MICB (MICB(s)), PNGase:F resistant MICB (MICB(r)), and deglycosylated MICB (deglycos) are indicated, as are approximate molecular weight markers (right). (TIF) [file ppat.1002362.s003.tif]

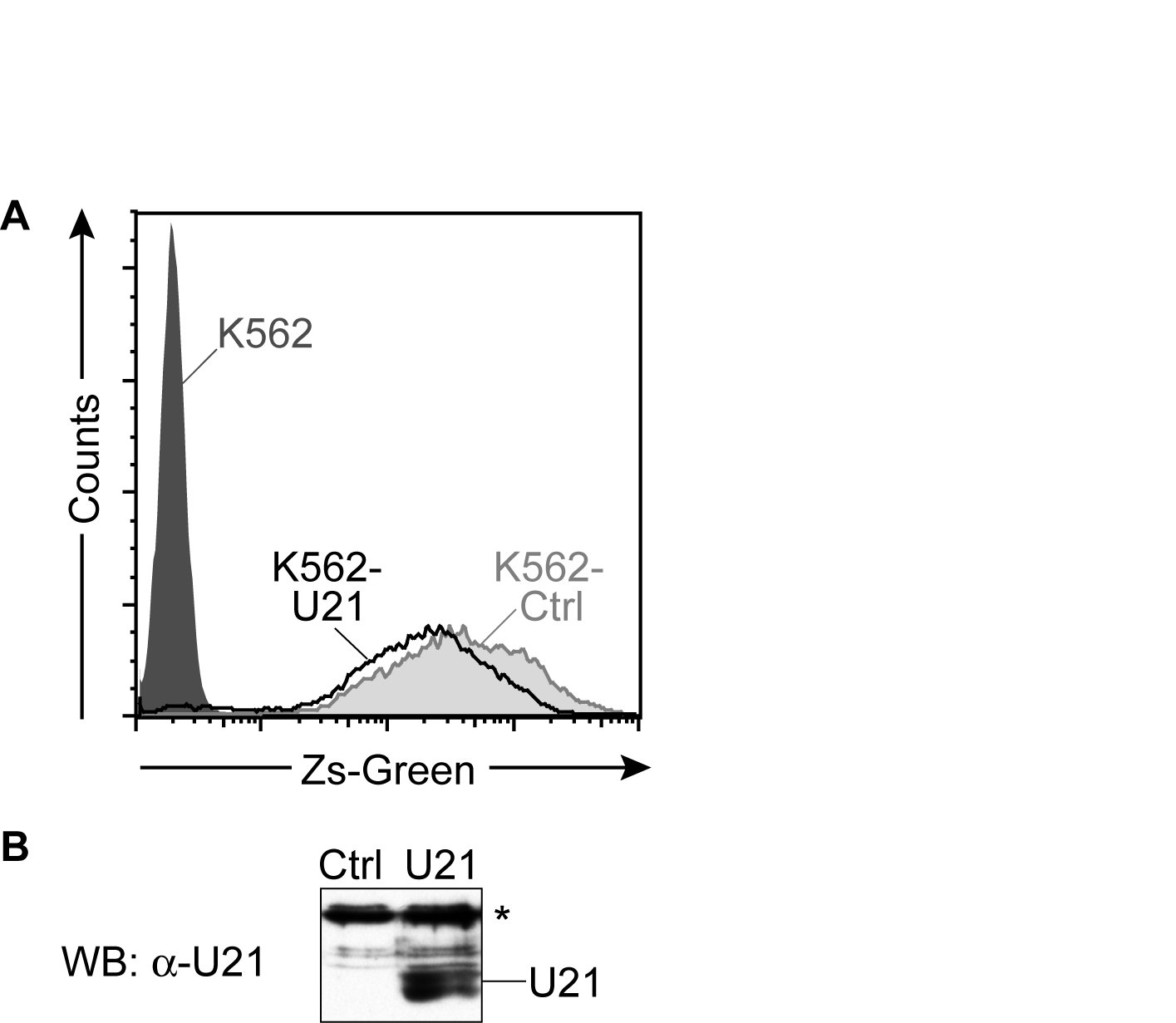

Supplement: Figure S4 — Expression of U21 in K562 cells. (A) Flow cytometric analysis of ZsGreen from K562 cells (dark gray solid), K562 cells expressing Zs-Green (light gray solid), or K562 cells expressing U21-IRES-ZsGreen (black line). (B) Cell lysates (30 µg) from ZsGreen- or U21-ZsGreen-expressing K562 cells were immunoblotted with an antibody directed against U21. A cross-reactive polypeptide recognized by the polyclonal U21 antibody serves as a loading control and is denoted with an asterisk. (TIF) [file ppat.1002362.s004.tif]

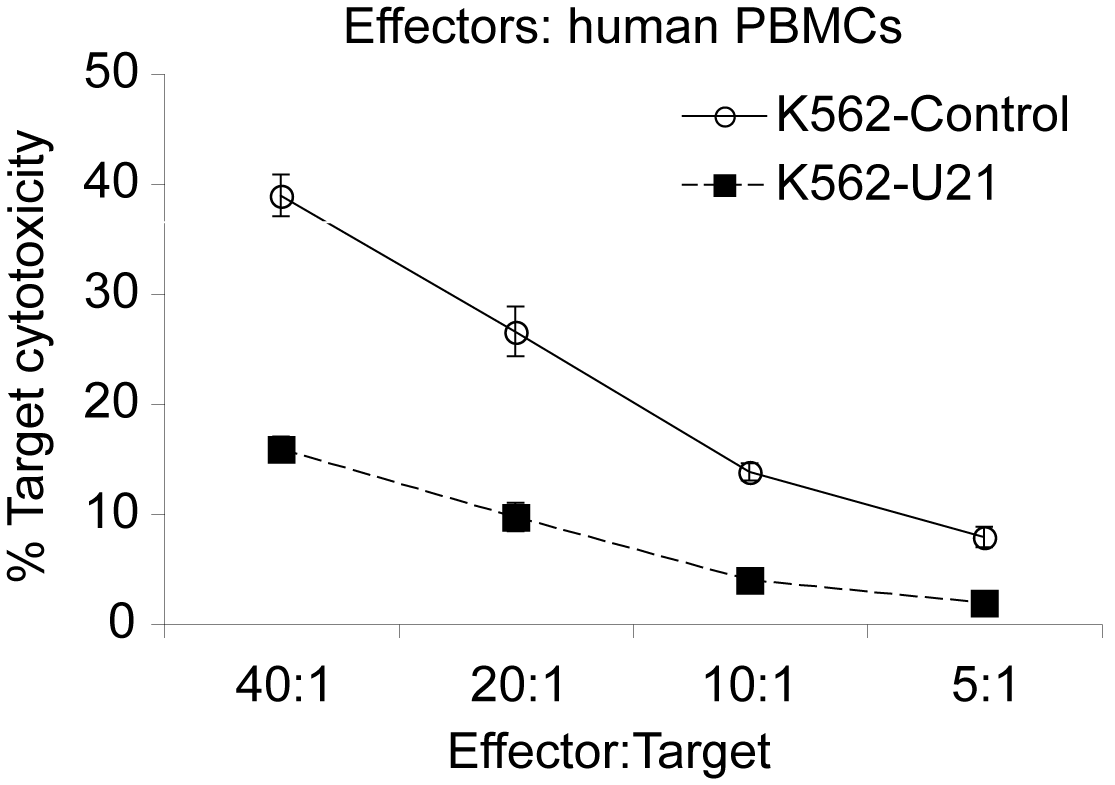

Supplement: Figure S5 — Expression of U21 in K562 cells reduces sensitivity to cytotoxicity mediated by human NK cells. Target K562 cells expressing ZsGreen or U21-ZsGreen were incubated in the presence of freshly isolated human peripheral blood mononuclear NK effector cells at the indicated E:T ratios. The graphs shown are single representative experiment performed in triplicate, and the error bars indicate the standard deviation between replicate samples (n = 1). (TIF) [file ppat.1002362.s005.tif]
